# A Prognostic Model Based on Nutritional Indexes for Patients With Pan‐Cancer: A Real‐World Cohort Study

**DOI:** 10.1002/cnr2.2121

**Published:** 2024-06-21

**Authors:** Lin Zheng, Qian‐Qian Yu, Wen‐Bin Ruan, Jin Chen, Qing‐Hua Deng, Ke Zhang, Xu‐Li Jiang, Wen‐Jun Jiang, Dan‐Na Cai, Chen‐Jie He, Yu‐Feng Wang, Shen‐Li Jiang, Rui‐Zhi Ye, Guang‐Xian You, Rong‐Biao Ying, Zhi‐Rui Zhou

**Affiliations:** ^1^ Department of Radiation Oncology Taizhou Cancer Hospital Wenling Zhejiang China; ^2^ Department of Radiotherapy, Affiliated Hangzhou Cancer Hospital Zhejiang University School of Medicine Hangzhou Zhejiang China; ^3^ Department of Medical Oncology Chengbei Branch of Taizhou Cancer Hospital Wenling Zhejiang China; ^4^ Department of Nursing Wenling First People's Hospital Wenling Zhejiang China; ^5^ Department of Nursing Taizhou Cancer Hospital Wenling Zhejiang China; ^6^ Department of Nursing Chengbei Branch of Taizhou Cancer Hospital Wenling Zhejiang China; ^7^ Department of Nutrition Chengbei Branch of Taizhou Cancer Hospital Wenling Zhejiang China; ^8^ Department of Surgical Oncology Taizhou Cancer Hospital Wenling Zhejiang China; ^9^ Radiation Oncology Center, Huashan Hospital, Shanghai Medical College Fudan University Shanghai China

**Keywords:** nutritional index, pan‐cancer, prognostic model, real‐world study

## Abstract

**Background:**

The aim was to identify the nutritional indexes, construct a prognostic model, and develop a nomogram for predicting individual survival probability in pan‐cancers.

**Methods:**

Nutritional indicators, clinicopathological characteristics, and previous major treatment details of the patients were collected. The enrolled patients were randomly divided into training and validation cohorts. Least absolute shrinkage and selection operator (Lasso) regression cross‐validation was used to determine the variables to include in the cox regression model. The training cohort was used to build the prediction model, and the validation cohort was used to further verify the discrimination, calibration, and clinical effectiveness of the model.

**Results:**

A total of 2020 patients were included. The median OS was 56.50 months (95% CI, 50.36–62.65 months). In the training cohort of 1425 patients, through Lasso regression cross‐validation, 13 characteristics were included in the model. Cox proportional hazards model was developed and visualized as a nomogram. The *C*‐indexes of the model for predicting 1‐, 3‐, 5‐, and 10‐year OS were 0.848, 0.826, 0.814, and 0.799 in the training cohort and 0.851, 0.819, 0.814, and 0.801 in the validation cohort. The model showed great calibration in the two cohorts. Patients with a score of less than 274.29 had a better prognosis (training cohort: HR, 6.932; 95% CI, 5.723–8.397; log‐rank *p* < 0.001; validation cohort: HR, 8.429; 95% CI, 6.180–11.497; log‐rank *p* < 0.001).

**Conclusion:**

The prognostic model based on the nutritional indexes of pan‐cancer can divide patients into different survival risk groups and performed well in the validation cohort.

## 1 Introduction

Malnutrition is a common problem among patients with cancers. It affects 40%–80% of patients during the course of their disease [1]. Malnutrition has an adverse effect on prognosis and increases the risk of disease‐related complications [2]. Therefore, the assessment of nutritional status should be a part of daily clinical practice and prognosis evaluation [2]. Patients with cancers may not always meet the traditional criteria for malnutrition, especially in the early stage of disease. However, they are still at considerable risk for malnutrition and related complications. At present, the commonly used nutritional screening tool for cancer patients is the Nutrition Risk Screening 2002 (NRS‐2002) [3, 4]. Some patients in the early stage of the disease have no nutritional risk according to the NRS‐2002; however, some of them could benefit from early nutritional support [5].

There are a variety of tools for screening and diagnosing malnutrition in the literature [6, 7, 8, 9, 10, 11, 12]. However, the diagnostic criteria for malnutrition lack validity, and the sensitivity and specificity of these tools do not meet the needs of clinicians and patients. Undeniably, the diagnosis of malnutrition in patients is a complex situation [13, 14, 15, 16], and different nutritional risk screening tools have their own characteristics and limitations. Cancer patients, especially elderly patients, are or may be at risk of developing malnutrition and its associated complications when admitted to the hospital [17], and they may not show clinical signs of malnutrition in the early stage of the disease. The NRS‐2002 alone cannot be used to identify all patients at nutritional risk, especially low‐risk patients. It is not possible to assess malnutrition based solely on a laboratory indicator.

A study by Jensen et al. [13], published in the *Journal of Clinical Nutrition*, provided some groundbreaking definitions of malnutrition, linking it to inflammation. A recent paper by Ruan et al. [18], published in *Frontiers in Nutrition*, also suggested that inflammation and malnutrition are closely related. A review by Jensen and Wheeler [19], in *Current Opinion in Critical Care*, suggested that serum albumin and prealbumin first reflect inflammation and then nutrition. Since inflammation reduces serum albumin and prealbumin levels, these indicators may not be effective in the assessment of nutritional risk under the NRS‐2002 tool for patients with low albumin or prealbumin levels. The presence of nutritional risk in patients is not the same as malnutrition. The benefits of early nutritional intervention may reflect its regulation of the systemic inflammatory response [19, 20].

Despite the recognition of the detrimental effects of malnutrition, traditional nutritional screening methods, such as the NRS‐2002, have limitations in accurately identifying patients at high risk of malnutrition and predicting their prognosis. Moreover, these methods may not fully capture the complex interplay between nutritional status, inflammation, and other clinical factors that influence cancer prognosis. Therefore, there is a need for more comprehensive and accurate prognostic models that integrate nutritional indicators with other relevant clinical parameters to better stratify patients according to their risk of poor outcomes. By developing a prognostic model based on nutritional indexes and other clinical factors, this study aims to address these gaps in existing approaches to nutritional assessment and prognostication in cancer patients. Fortunately, it is suggested by our results that the prognostic prediction model can well stratify the prognosis of cancer patients.

## 2 Materials and Methods

### 2.1 Patients and Datasets

Based on the real‐world settings of clinical diagnosis and treatment, patients admitted to the Department of Radiotherapy and Chemotherapy of Taizhou Cancer Hospital from January 1, 2017 to July 1, 2020 were included in this retrospective cohort study. The study was approved by the Ethics Committee of Taizhou Cancer Hospital. Because this was a retrospective study, all patients were exempted from providing informed consent for the study. The patients received antitumor therapy for their diagnosed cancer. The inclusion and exclusion criteria were as follows.

Inclusion criteria: (1) age ≥18 years old; (2) at least one hospitalization during the course of the disease; (3) histopathological diagnosis or cytological diagnosis must be obtained; (4) received at least one kind of antitumor therapy (surgery, radiotherapy, chemotherapy, targeted therapy, or immunotherapy); (5) meet the requirements for nutritional index assessment; (6) complete clinical medical history records; and (7) complete follow‐up record available.

Exclusion criteria: (1) patients with mental disorders; (2) simultaneous multicentric malignant tumors; (3) unavailable histopathological or cytological diagnosis; (4) lack of relevant predictive indicators (patients who lacked routine blood and biochemical examination results and those lost to follow‐up within 1 month after treatment); (5) lack of routine blood examinations once a week and biochemical examinations (mainly including liver and kidney function) twice a week during antitumor therapy or within 1 month after treatment; (6) patients who could not be contacted at the first follow‐up conducted within 1 month after treatment (patients who could not be contacted after the initial follow‐up were treated as “censored”) and (7) Combined with other nutritional diseases such as malabsorption and metabolic abnormalities.

The clinicopathological data were obtained from the electronic medical record system of Taizhou Cancer Hospital. The last follow‐up date was April 1, 2022. This study followed the Transparent Reporting of a Multivariable Prediction Model for Individual Prognosis or Diagnosis (TRIPOD) reporting guidelines for prognostic studies [21].

### 2.2 Clinicopathological Variables and Outcome

Based on a literature review and clinical practice experience [13, 14, 17, 19, 22, 23, 24, 25], considering clinical accessibility, the following routine clinicopathological variables of the patients were collected: age, sex, ECOG performance status, height, baseline weight (weight at diagnosis of disease), BMI, weight loss within the last 3 months, changes in food intake within the last week, NRS‐2002, C‐reactive protein (CRP), procalcitonin (PCT), infection, prealbumin, albumin, total protein, retinol‐binding protein, cholesterol, white blood cell count, hemoglobin, platelet count, lymphocyte count, bone marrow suppression, liver function classification, cancer site, underlying diseases (such as hypertension, diabetes, cardiovascular or cerebrovascular diseases, chronic hepatitis or liver cirrhosis, chronic obstructive pulmonary disease, and history of other cancers) and emerging diseases during hospitalization (mainly including infection, complications of radiotherapy and chemotherapy, digestive tract obstruction, digestive tract bleeding, digestive tract perforation, hydrothorax or ascites, cardiopulmonary insufficiency, deep vein thrombosis, etc.), tumor fever, and T, N, and M stages.

Toxicity was graded by using the Common Terminology Criteria for Adverse Events (CTCAE), version 4.03. For patients who were hospitalized repeatedly, the worst level of indicators in the previous examination was collected. Clinical stage and baseline weight were collected when the disease was diagnosed. However, the other data were collected when oncologists treated the patients.

The end point of the study was overall survival (OS), defined as the time interval between the patient's pathological diagnosis and death or the last follow‐up.

### 2.3 Statistical Analysis

Statistical processing of the results was performed using R software (version 4.2.1; http://www.r‐project.org) and IBM SPSS 25.0 (IBM Corp., Armonk, NY, USA). The R packages mainly used included rms, glmnet, and pec. Categorical variables are expressed as percentages. Continuous variables are expressed as the mean ± standard deviation, median and range, or median and interquartile range (IQR), as appropriate. The Kolmogorov–Smirnov test was used as the normality test, and the results of this test were combined with the results of the histogram, *Q*–*Q* plot, skewness coefficient, and kurtosis coefficient results. Comparisons between two groups were assessed with the chi‐square test or Fisher's exact test for categorical variables and the *t*‐test or Wilcoxon rank‐sum test for continuous variables, as appropriate. The ordinal data were treated as continuous variables in the multivariate analysis modeling approach. A *p* < 0.05 was considered statistically significant.

The dataset was randomly divided into a training cohort and a validation cohort at a ratio of 7:3. The training cohort was used for cross‐validation of Lasso regression using *λ*_min_ and *λ*_min_ + 1 SE to determine the characteristics to include in the final Cox model [26]. Lasso regression is a method for variable selection that penalizes model coefficients, effectively identifying the most important variables in cases with numerous predictors. During cross‐validation, the dataset is divided into subsets, and each model training selects different subsets for validation, preventing overfitting to specific datasets and improving model generalization.

Multivariable Cox proportional hazards regression was used in the training cohort to construct the prediction model, which was visualized as a nomogram, and the discrimination and calibration of the model were further verified in the validation cohort. The risk scores of the patients in the training and validation cohorts were calculated based on the nomogram of the Cox model. Then, the patients were divided into a low‐risk group and a high‐risk group according to their score. The survival curves of the two groups were generated by the Kaplan–Meier method and compared with the log‐rank test.

## 3 Results

### 3.1 Baseline Characteristics

A total of 2020 patients were enrolled in our study. Among them, 51.68% died. The median follow‐up duration was 33.48 months (IQR, 15.79–56.73), and the median OS was 56.50 months (95% CI, 50.36–62.65). According to the anatomical location, the cancers were divided into 12 types. Respiratory system cancers were the most common, accounting for 26.4%, followed by breast cancer (14.8%). There were 1073 male patients and 947 female patients. The average age was 61.89 years old. The average BMI of the overall sample was 22.05 kg/m^2^, of which 18.5% of patients had BMI ≥ 25 kg/m^2^ and 14.9% of patients had BMI <18.5 kg/m^2^. Patients with an NRS score ≥3 accounted for 37.5%. The proportions of patients with prealbumin and albumin levels below normal values were 53.9% and 59.6%, respectively. Patients with ECOG scores <2 accounted for 50.9%. Patients with Stage I/II disease accounted for 37.7%, and the remaining 62.3% of patients had Stage III/IV disease. Table 1 provides more detailed patient characteristics of the full dataset, training cohort, and validation cohort.

**TABLE 1:** Demographics and clinicopathologic characteristics of patients with pan‐cancer.

Demographic or clinical characteristic	Overall (*n* = 2020)	Training cohort (*n* = 1425)	Validation cohort (*n* = 595)	*p*
Sex (%)				0.728
Male	1073 (53.1)	761 (53.4)	312 (52.4)	
Female	947 (46.9)	664 (46.6)	283 (47.6)	
Age, mean (SD), years	61.89 (11.72)	61.68 (12.01)	62.41 (11.00)	0.203
Height, mean (SD), m	162.64 (7.48)	162.70 (7.40)	162.48 (7.64)	0.547
Baseline weight, mean (SD), kg	59.56 (10.96)	59.66 (10.92)	59.32 (11.05)	0.532
BMI, mean (SD), kg/m^2^	22.05 (3.55)	22.09 (3.57)	21.96 (3.50)	0.498
BMI grade (%)				0.084
30–40 kg/m^2^	42 (2.2)	31 (2.3)	11 (1.9)	
25–29.9 kg/m^2^	310 (16.3)	225 (16.9)	85 (15.0)	
18.5–24.9 kg/m^2^	1263 (66.5)	883 (66.2)	380 (67.3)	
17–18.4 kg/m^2^	153 (8.1)	113 (8.5)	40 (7.1)	
16–16.9 kg/m^2^	61 (3.2)	33 (2.5)	28 (5.0)	
<16 kg/m^2^	69 (3.6)	48 (3.6)	21 (3.7)	
Weight loss (median [range]), kg	0.00 [0.00, 20.00]	0.00 [0.00, 15.00]	0.00 [0.00, 15.00]	0.602
Weight loss grade (%)				0.508
None	1524 (80.7)	1080 (81.1)	444 (79.6)	
<5%	63 (3.3)	43 (3.2)	20 (3.6)	
5%–10%	213 (11.3)	142 (10.7)	71 (12.7)	
>10%	89 (4.7)	66 (5.0)	23 (4.1)	
Food intake reduction grade (%)				0.808
<25%	1528 (76.0)	1075 (75.8)	453 (76.5)	
25%–50%	151 (7.5)	110 (7.8)	41 (6.9)	
51%–75%	220 (10.9)	158 (11.1)	62 (10.5)	
76%–100%	112 (5.6)	76 (5.4)	36 (6.1)	
NRS‐2002 (%), points				0.523
1	954 (47.5)	679 (47.8)	275 (46.6)	
2	302 (15.0)	206 (14.5)	96 (16.3)	
3	204 (10.1)	154 (10.8)	50 (8.5)	
4	378 (18.8)	264 (18.6)	114 (19.3)	
5	164 (8.2)	111 (7.8)	53 (9.0)	
6	8 (0.4)	6 (0.4)	2 (0.3)	
Underlying disease (%)				0.890
No	1158 (57.3)	815 (57.2)	343 (57.6)	
Yes	862 (42.7)	610 (42.8)	252 (42.4)	
Emerging disease (%)				0.649
No	1114 (55.1)	791 (55.5)	323 (54.3)	
Yes	906 (44.9)	634 (44.5)	272 (45.7)	
ECOG performance status (%), points				0.742
0	212 (10.5)	152 (10.7)	60 (10.1)	
1	812 (40.4)	562 (39.6)	250 (42.2)	
2	505 (25.1)	356 (25.1)	149 (25.2)	
3	331 (16.5)	237 (16.7)	94 (15.9)	
4	151 (7.5)	112 (7.9)	39 (6.6)	
Tumor fever (%)				0.884
No	1989 (98.5)	1404 (98.5)	585 (98.3)	
Yes	31 (1.5)	21 (1.5)	10 (1.7)	
Hospitalization frequency (median [IQR]), times	3.00 [1.00, 7.00]	2.00 [1.00, 6.00]	3.00 [1.00, 7.00]	0.083
CRP (median [IQR]), mg/L	9.40 [1.62, 46.08]	9.36 [1.47, 46.60]	9.50 [1.85, 44.94]	0.655
PCT (median [IQR]), ng/mL	0.06 [0.03, 0.14]	0.06 [0.03, 0.14]	0.05 [0.02, 0.16]	0.735
Total protein, mean (SD), g/L	68.83 (8.42)	68.80 (8.50)	68.92 (8.26)	0.757
Prealbumin (median [IQR]), mg/dL	160.00 [91.00, 216.00]	159.00 [91.50, 216.00]	162.00 [91.00, 215.00]	0.674
Albumin (median [IQR]), g/L	38.40 [32.92, 42.40]	38.30 [32.90, 42.30]	38.55 [33.00, 42.77]	0.485
Retinol binding protein, mean (SD), mg/L	30.23 (14.88)	30.19 (14.99)	30.33 (14.62)	0.848
Cholesterol, mean (SD), mmol/L	5.54 (1.51)	5.52 (1.49)	5.58 (1.54)	0.381
Cancer site (%)				0.353
Head and neck	192 (9.5)	127 (8.9)	65 (10.9)	
Intracranial	28 (1.4)	18 (1.3)	10 (1.7)	
Respiratory	534 (26.4)	379 (26.6)	155 (26.1)	
Breast	298 (14.8)	213 (14.9)	85 (14.3)	
Gastroesophageal	158 (7.8)	108 (7.6)	50 (8.4)	
Pancreas	43 (2.1)	33 (2.3)	10 (1.7)	
Liver, cholecyst, and intrahepatic bile ducts	121 (6.0)	82 (5.8)	39 (6.6)	
Colorectal	222 (11.0)	159 (11.2)	63 (10.6)	
Hematologic and lymphoma	90 (4.5)	58 (4.1)	32 (5.4)	
Bone and soft tissue	21 (1.0)	19 (1.3)	2 (0.3)	
Genitourinary	239 (11.8)	171 (12.0)	68 (11.4)	
Other cancers	74 (3.7)	58 (4.1)	16 (2.7)	
T stage (%)				0.288
0	7 (0.5)	3 (0.3)	4 (1.1)	
1	295 (23.0)	219 (23.8)	76 (21.0)	
2	459 (35.8)	334 (36.3)	125 (34.5)	
3	273 (21.3)	190 (20.7)	83 (22.9)	
4	247 (19.3)	173 (18.8)	74 (20.4)	
N stage (%)				0.146
0	472 (34.1)	353 (35.7)	119 (30.1)	
1	364 (26.3)	247 (25.0)	117 (29.5)	
2	361 (26.1)	259 (26.2)	102 (25.8)	
3	188 (13.6)	130 (13.1)	58 (14.6)	
M stage (%)				0.534
0	1157 (70.5)	831 (71.4)	326 (68.5)	
1	480 (29.3)	331 (28.4)	149 (31.3)	
2	1 (0.1)	1 (0.1)	0 (0.0)	
3	2 (0.1)	1 (0.1)	1 (0.2)	
Clinical stages (%)				0.099
I	291 (14.4)	223 (15.6)	68 (11.4)	
II	470 (23.3)	328 (23.0)	142 (23.9)	
III	623 (30.8)	436 (30.6)	187 (31.4)	
IV	636 (31.5)	438 (30.7)	198 (33.3)	
Infection (%)				0.991
No	1618 (80.1)	1142 (80.1)	476 (80.0)	
Yes	402 (19.9)	283 (19.9)	119 (20.0)	
Lymphocyte count (median [IQR]), ×10^9^/L	0.98 [0.67, 1.39]	0.99 [0.67, 1.43]	0.94 [0.66, 1.30]	0.044
White blood cell count (median [IQR]), ×10^9^/L	3.90 [2.70, 5.50]	3.90 [2.70, 5.50]	3.90 [2.60, 5.40]	0.522
White blood cell suppression grade (%)				0.991
Normal	999 (50.1)	709 (50.2)	290 (49.7)	
I	416 (20.8)	295 (20.9)	121 (20.8)	
II	352 (17.6)	250 (17.7)	102 (17.5)	
III	182 (9.1)	127 (9.0)	55 (9.4)	
IV	47 (2.4)	32 (2.3)	15 (2.6)	
Neutrophils count, (median [IQR]), ×10^9^/L	2.47 [1.51, 3.68]	2.49 [1.53, 3.73]	2.41 [1.50, 3.67]	0.550
Neutrophils suppression grade (%)				0.159
Normal	1314 (65.8)	937 (66.3)	377 (64.7)	
I	205 (10.3)	140 (9.9)	65 (11.1)	
II	219 (11.0)	159 (11.3)	60 (10.3)	
III	170 (8.5)	109 (7.7)	61 (10.5)	
IV	88 (4.4)	68 (4.8)	20 (3.4)	
Hemoglobin, mean (SD), g/L	108.54 (24.96)	108.37 (25.42)	108.96 (23.85)	0.630
Hemoglobin suppression grade (%)				0.059
Normal	1072 (53.5)	767 (54.1)	305 (52.0)	
I	388 (19.4)	260 (18.3)	128 (21.8)	
II	340 (17.0)	236 (16.7)	104 (17.7)	
III	179 (8.9)	132 (9.3)	47 (8.0)	
IV	24 (1.2)	22 (1.6)	2 (0.3)	
Platelet count (median [IQR]), ×10^9^/L	159.00 [103.00, 209.00]	159.00 [102.00, 208.00]	159.00 [108.00, 210.50]	0.341
Platelet suppression grade (%)				0.659
Normal	1549 (77.3)	1087 (76.7)	462 (78.8)	
I	160 (8.0)	113 (8.0)	47 (8.0)	
II	111 (5.5)	85 (6.0)	26 (4.4)	
III	103 (5.1)	73 (5.2)	30 (5.1)	
IV	80 (4.0)	59 (4.2)	21 (3.6)	
ALT (median [IQR]), IU/L	23.50 [14.60, 46.65]	23.50 [14.50, 46.25]	23.90 [15.10, 47.90]	0.725
AST (median [IQR]), IU/L	28.10 [20.80, 46.18]	27.70 [20.60, 45.80]	29.20 [21.25, 47.05]	0.207
Liver function classification (%)				0.224
Normal	1163 (58.3)	843 (59.7)	320 (54.9)	
I	602 (30.2)	405 (28.7)	197 (33.8)	
II	142 (7.1)	99 (7.0)	43 (7.4)	
III	77 (3.9)	57 (4.0)	20 (3.4)	
IV	10 (0.5)	7 (0.5)	3 (0.5)	

Abbreviations: ALT, alanine aminotransferase; AST, glutamic oxalacetic transaminase; BMI, body mass index; CRP, C‐reactive protein; ECOG, Eastern Cooperative Oncology Group; IQR, interquartile range; NRS, nutritional risk screening; PCT, procalcitonin; SD, standard deviation.

### 3.2 Characteristic Selection

**FIGURE 1:**
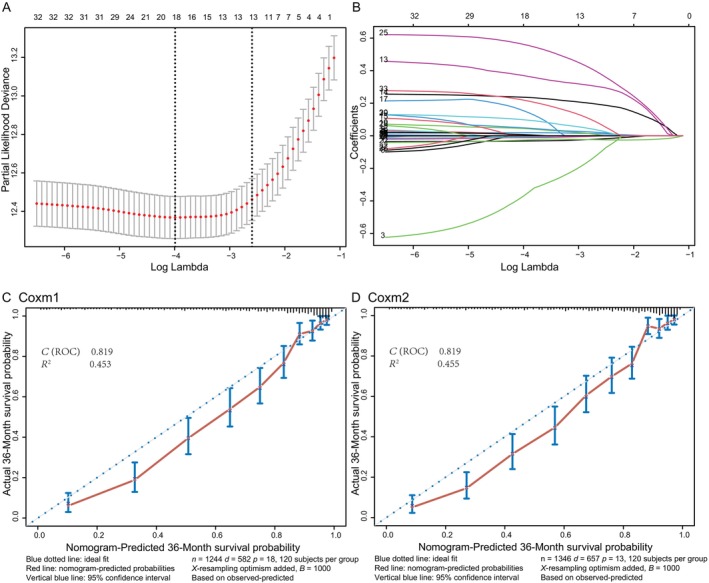
Lasso Cox regression model for variable selection and calibration curves of OS prediction in the training cohort. (A) The minimum *λ* (*λ*_min_) and the 1 standard error of the minimum *λ* (*λ*_min_ + 1 SE) were selected by the 10‐fold cross‐validation method. The partial likelihood deviance was plotted versus log(*λ*). Dotted vertical lines were drawn at the optimal values by using *λ*_min_ and *λ*_min_ + 1 SE. According to 10‐fold cross‐validation, log(*λ*_min_) = −3.991645 and log(*λ*_min_ + 1 SE) = −2.596139 were selected for the model, and 18 and 13 characteristics were screened. (B) Lasso coefficient profiles of the 33 characteristics. A coefficient profile plot was produced against the log(*λ*) sequence. (C) In the training cohort, calibration curves were used to evaluate the effectiveness of the nomogram in predicting OS based on Coxm1. (D) In the training cohort, the nomogram was used to evaluate the effectiveness of predicting OS based on Coxm2. CI, confidence interval; ROC, receiver operating characteristic; OS, overall survival.

First, the Lasso cross‐validation regression method was used in the training cohort of 1425 patients to determine the penalty coefficient *λ*. Subsequently, *λ*_min_ (0.0184693) and *λ*_min_ + 1 SE (0.07456087) were introduced into the Lasso regression model. This process screened out 18 and 13 characteristics with nonzero regression coefficients, respectively (Figure 1A,B). Then, *λ*_min_ and *λ*_min_ + 1 SE were selected to construct the prediction models using Cox regression respectively; that is, a model with 18 characteristics named “Coxm1” and a model with 13 characteristics named “Coxm2” were constructed. Meanwhile, the corresponding regression coefficients were calculated. The Coxm2 was finally used after evaluation and validation, following the principle of optimal clinical use. The 13 characteristics included in Coxm2 were as follows: sex, age, baseline weight, food intake reduction grade, emerging disease, ECOG performance status, hospitalization frequency, prealbumin, albumin, clinical stage, hemoglobin suppression grade, platelet suppression grade, and liver function classification.

### 3.3 Nutritional Indicators Related to the Prognosis of Cancers

As expected, subjects with a worse prognosis had a significantly lower baseline weight (HR, 0.984; 95% CI, 0.976–0.991; *p* < 0.0001), lower prealbumin level (HR, 0.997; 95% CI, 0.995–0.998; *p* < 0.0001), lower albumin level (HR, 0.98; 95% CI, 0.962–0.998; *p* = 0.027), more reduced food intake in the last week than usual (HR, 1.087; 95% CI, 1.001–1.181; *p* = 0.047), and abnormal liver function (HR, 1.216; 95% CI, 1.109–1.332; *p* < 0.0001), according to the multivariable analysis results.

It was also found that patients with a worse prognosis showed more hemoglobin suppression (HR, 1.507; 95% CI, 1.423–1.596; *p* < 0.0001) and platelet suppression (HR, 1.264; 95% CI, 1.194–1.194; *p* < 0.0001) in univariable analysis. In multivariable regression analysis, there was no statistical significance. However, according to clinical practice, considering that these factors also have a great influence on the prognosis of patients, they were included in the final Cox model.

### 3.4 Univariable and Multivariable Analyses and Nomogram Construction

**FIGURE 2:**
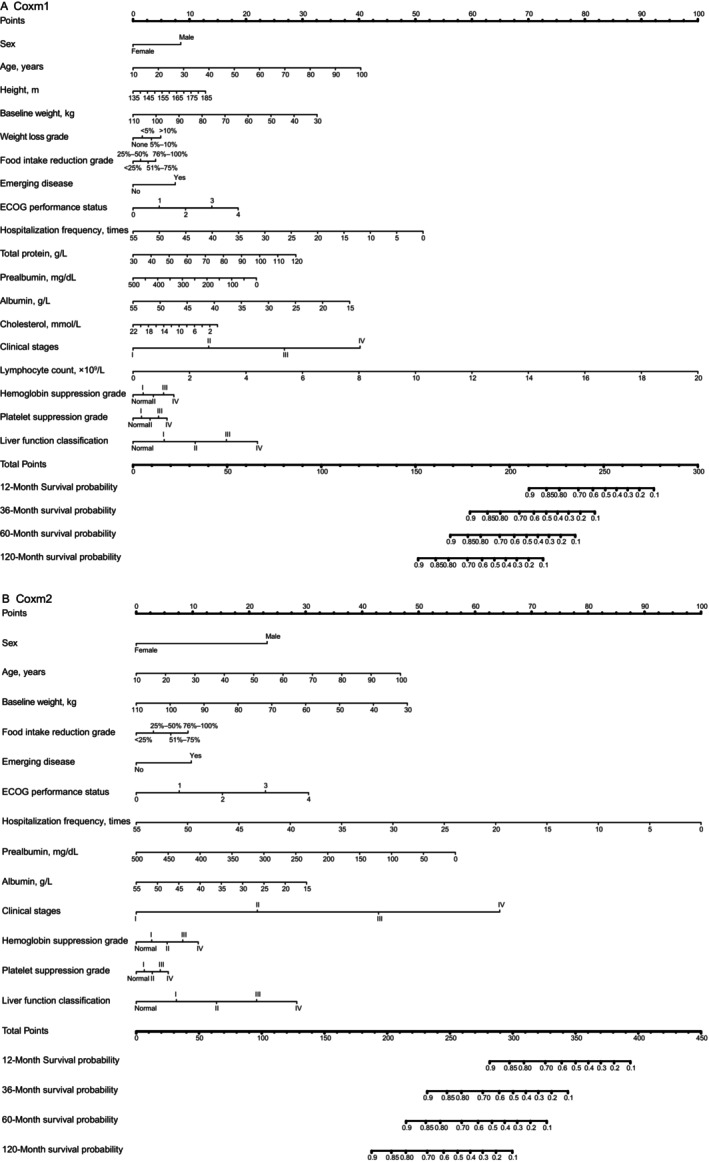
Cox model for OS prediction based on nutrition indexes in the training cohort. The instructions of the nomogram are as follows: find the position of each variable on the corresponding axis, and draw a vertical line on this position to obtain the score of the variable. Next, add the scores of all variables to calculate the total score, find the position of the total score on the total points axis, and then draw a vertical line to determine the survival probability on the nomogram. (A) Nomogram for predicting 1‐, 3‐, 5‐, and 10‐year OS based on 18 characteristics: “Coxm1.” (B) Nomogram for predicting 1‐, 3‐, 5‐, and 10‐year OS based on 13 characteristics: “Coxm2.” ECOG, Eastern Cooperative Oncology Group.

The analysis results of the univariable Cox proportional hazards model in the training cohort are given in Table 2. The characteristics selected by Lasso regression cross‐validation in the training cohort were included in multivariable analysis. In the Coxm2 model, multivariable analysis showed that there were 11 independent prognostic variables: sex, age, baseline weight, food intake reduction grade, emerging disease, ECOG performance status, hospitalization frequency, prealbumin, albumin, clinical stage, and liver function classification. By comparison, the prognosis of female patients (HR, 0.528; 95% CI, 0.444–0.629; *p* < 0.0001), younger patients (HR, 1.014; 95% CI, 1.007–1.022; *p* < 0.0001), patients with a higher baseline weight (HR, 0.984; 95% CI, 0.976–0.991; *p* < 0.0001), patients with a higher prealbumin level (HR, 0.997; 95% CI, 0.995–0.998; *p* < 0.0001), patients with a higher albumin level (HR, 0.98; 95% CI, 0.962–0.998; *p* = 0.027), patients with repeated hospitalization (HR, 0.951; 95% CI, 0.937–0.966; *p* < 0.0001) (which may indicate patients actively undergoing antitumor therapy), and patients with an earlier clinical stage (HR, 1.804; 95% CI, 1.65–1.972; *p* < 0.0001) had a better prognosis. Those who had a worse prognosis had a more reduced food intake in the last week than usual (HR, 1.087; 95% CI, 1.001–1.181; *p* = 0.047), emerging disease (HR, 1.308; 95% CI, 1.091–1.568; *p* = 0.004), an increased ECOG performance score (HR, 1.233; 95% CI, 1.13–1.346; *p* < 0.0001), and abnormal liver function (HR, 1.216; 95% CI, 1.109–1.332; *p* < 0.0001). The analysis results of the multivariable Cox proportional hazards model in the training cohort are given in Table 3. The multivariable analysis results were visualized as a nomogram (Figure 2A,B).

**TABLE 2:** Univariable cox regression analyses based on nutritional indicators in the training cohort.

Variable	*β*	SE	*p*	HR (95% CI)
Sex	−0.784	0.079	<0.0001	0.457 (0.391–0.533)
Age	0.033	0.003	<0.0001	1.034 (1.027–1.040)
Height	0.033	0.005	<0.0001	1.034 (1.023–1.044)
Baseline weight	−0.025	0.004	<0.0001	0.975 (0.968–0.982)
BMI	−0.121	0.012	<0.0001	0.886 (0.866–0.906)
BMI grade	0.321	0.036	<0.0001	1.379 (1.284–1.481)
Weight loss	0.087	0.012	<0.0001	1.091 (1.065–1.117)
Weight loss grade	0.284	0.039	<0.0001	1.328 (1.231–1.433)
Food intake reduction grade	0.489	0.035	<0.0001	1.631 (1.524–1.746)
NRS‐2002	0.344	0.024	<0.0001	1.411 (1.346–1.479)
Underlying disease	0.279	0.074	<0.0001	1.322 (1.143–1.530)
Emerging disease	1.141	0.078	<0.0001	3.131 (2.687–3.649)
ECOG performance status	0.596	0.032	<0.0001	1.815 (1.705–1.933)
Tumor fever	0.768	0.246	0.002	2.156 (1.332–3.491)
Hospitalization frequency	−0.033	0.007	<0.0001	0.968 (0.954–0.982)
CRP	0.006	0.000	<0.0001	1.006 (1.005–1.007)
PCT	0.011	0.006	0.060	1.011 (1.000–1.022)
Total protein	−0.050	0.004	<0.0001	0.951 (0.944–0.959)
Prealbumin	−0.010	0.001	<0.0001	0.990 (0.989–0.991)
Albumin	−0.106	0.005	<0.0001	0.899 (0.890–0.909)
Retinol binding protein	−0.041	0.003	<0.0001	0.959 (0.954–0.965)
Cholesterol	−0.172	0.029	<0.0001	0.842 (0.795–0.891)
Cancer site			<0.0001	
Pancreas				Ref
Head and neck	−1.710	0.226	<0.0001	0.181 (0.116–0.282)
Intracranial	−1.820	0.397	<0.0001	0.162 (0.074–0.353)
Respiratory	−0.804	0.190	<0.0001	0.448 (0.308–0.650)
Breast	−3.018	0.270	<0.0001	0.049 (0.029–0.083)
Gastroesophageal	−1.059	0.219	<0.0001	0.347 (0.226–0.533)
Liver, cholecyst, and intrahepatic bile ducts	−0.493	0.217	0.023	0.611 (0.399–0.934)
Colorectal	−1.518	0.215	<0.0001	0.219 (0.144–0.334)
Hematologic and lymphoma	−1.629	0.270	<0.0001	0.196 (0.116–0.333)
Bone and soft tissue	−0.722	0.322	0.025	0.486 (0.258–0.914)
Genitourinary	−2.089	0.227	<0.0001	0.124 (0.079–0.193)
Other cancers	−1.184	0.251	<0.0001	0.306 (0.187–0.501)
Clinical stages	0.710	0.042	<0.0001	2.033 (1.872–2.209)
Infection	0.953	0.079	<0.0001	2.593 (2.221–3.027)
Lymphocyte count	−0.341	0.071	<0.0001	0.711 (0.619–0.817)
White blood cell count	0.046	0.008	<0.0001	1.047 (1.031–1.064)
White blood cell suppression grade	0.101	0.033	0.002	1.106 (1.036–1.180)
Neutrophils count	0.059	0.008	<0.0001	1.060 (1.044–1.078)
Neutrophils suppression grade	0.077	0.030	0.011	1.080 (1.018–1.146)
Hemoglobin	−0.020	0.001	<0.0001	0.980 (0.978–0.983)
Hemoglobin suppression grade	0.410	0.029	<0.0001	1.507 (1.423–1.596)
Platelet count	−0.003	0.000	<0.0001	0.997 (0.996–0.998)
Platelet suppression grade	0.234	0.029	<0.0001	1.264 (1.194–1.337)
ALT	0.001	0.000	<0.0001	1.001 (1.001–1.002)
AST	0.000	0.000	<0.0001	1.000 (1.000–1.001)
Liver function classification	0.392	0.040	<0.0001	1.480 (1.369–1.601)

Abbreviations: ALT, alanine aminotransferase; AST, glutamic oxalacetic transaminase; BMI, body mass index; CRP, C‐reactive protein; ECOG, Eastern Cooperative oncology Group; NRS, nutritional risk screening; PCT, procalcitonin.

**TABLE 3:** Multivariable cox regression analyses based on nutritional indicators in the training cohort.

Variable	Coxm1				Coxm2			
	*β*	SE	*p*	HR (95% CI)	*β*	SE	*p*	HR (95% CI)
Sex	−0.393	0.129	0.002	0.675 (0.524–0.870)	−0.638	0.089	<0.0001	0.528 (0.444–0.629)
Age	0.021	0.004	<0.0001	1.021 (1.013–1.029)	0.014	0.004	<0.0001	1.014 (1.007–1.022)
Height	0.012	0.009	0.181	1.012 (0.994–1.030)				
Baseline weight	−0.019	0.005	<0.0001	0.981 (0.972–0.990)	−0.017	0.004	<0.0001	0.984 (0.976–0.991)
Weight loss grade	0.076	0.044	0.085	1.079 (0.990–1.176)				
Food intake reduction grade	0.062	0.048	0.195	1.064 (0.969–1.168)	0.084	0.042	0.047	1.087 (1.001–1.181)
Emerging disease	0.348	0.097	<0.0001	1.416 (1.169–1.714)	0.268	0.093	0.004	1.308 (1.091–1.568)
ECOG performance status	0.216	0.047	<0.0001	1.241 (1.131–1.362)	0.210	0.045	<0.0001	1.233 (1.13–1.346)
Hospitalization frequency	−0.043	0.008	<0.0001	0.958 (0.942–0.973)	−0.050	0.008	<0.0001	0.951 (0.937–0.966)
Total protein	0.015	0.006	0.022	1.015 (1.002–1.028)				
Prealbumin	−0.002	0.001	0.026	0.998 (0.996–1.000)	−0.003	0.001	<0.0001	0.997 (0.995–0.998)
Albumin	−0.045	0.012	<0.0001	0.956 (0.934–0.979)	−0.021	0.009	0.027	0.98 (0.962–0.998)
Cholesterol	−0.032	0.028	0.255	0.969 (0.918–1.023)				
Clinical stages	0.623	0.049	<0.0001	1.864 (1.692–2.053)	0.590	0.046	<0.0001	1.804 (1.650–1.972)
Lymphocyte count	0.232	0.076	0.002	1.262 (1.088–1.463)				
Hemoglobin suppression grade	0.084	0.046	0.070	1.088 (0.993–1.191)	0.076	0.041	0.064	1.079 (0.996–1.168)
Platelet suppression grade	0.070	0.038	0.069	1.072 (0.995–1.156)	0.039	0.035	0.268	1.040 (0.970–1.114)
Liver function classification	0.256	0.052	<0.0001	1.292 (1.166–1.431)	0.195	0.047	<0.0001	1.216 (1.109–1.332)

Abbreviation: ECOG, Eastern Cooperative Oncology Group.

### 3.5 Verification in the Training and Validation Cohorts

The bootstrap method with 1000 resampling was used to verify the model in the validation cohort. The nomogram showed great discrimination in predicting OS, with *C*‐indexes for Coxm1 and Coxm2 of 0.819 (95% CI, 0.803–0.835) and 0.819 (95% CI, 0.803–0.835), respectively. In addition, the calibration curve showed good agreement between the predicted survival rate and the actual observed survival rate (Figure 1C,D).

**FIGURE 3:**
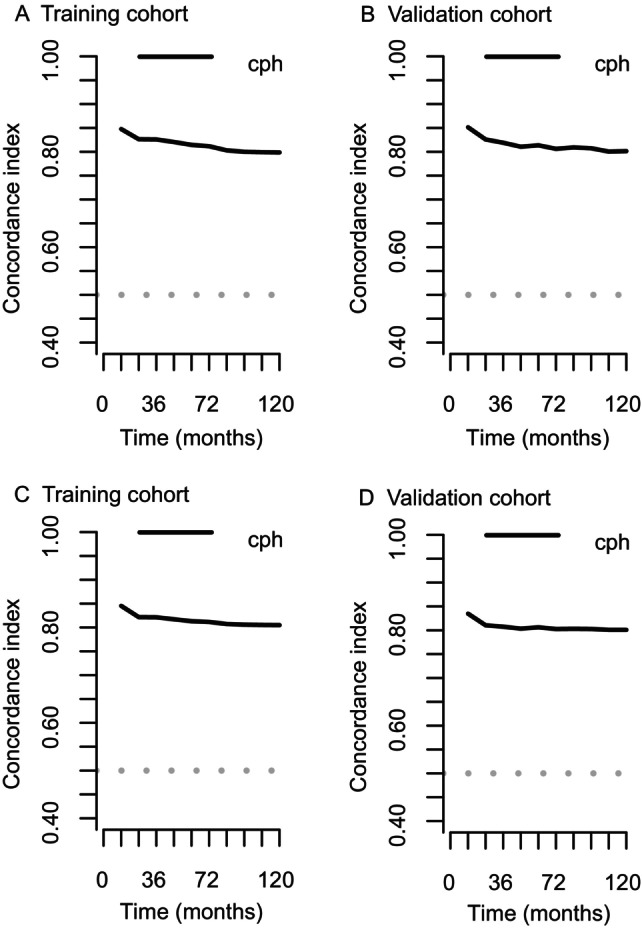
*C*‐indexes based on Coxm2 at various time points in the training and validation cohorts. (A) *C*‐indexes at various time points in the training cohort. (B) *C*‐indexes at various time points in the validation cohort. (C) Resampling *C*‐indexes at various time points in the training cohort. (D) Resampling *C*‐indexes at various time points in the validation cohort. *C*‐index, concordance index.

The Coxm2 model was chosen for further evaluation in the training cohort. The *C*‐indexes at 1, 3, 5, and 10 years were 0.848, 0.826, 0.814, and 0.799, respectively; the resampling *C*‐indexes were 0.845, 0.821, 0.813, and 0.805, respectively. The *C*‐indexes of Coxm2 at 1, 3, 5, and 10 years in the validation cohort were 0.851, 0.819, 0.814, and 0.801, respectively, and those of the resampling were 0.835, 0.807, 0.806, and 0.801, respectively. The *C*‐indexes of Coxm2 at various time points in the training and validation cohorts are shown in Figure 3A,B, and the resampling *C*‐indexes are shown in Figure 3C,D.

**FIGURE 4:**
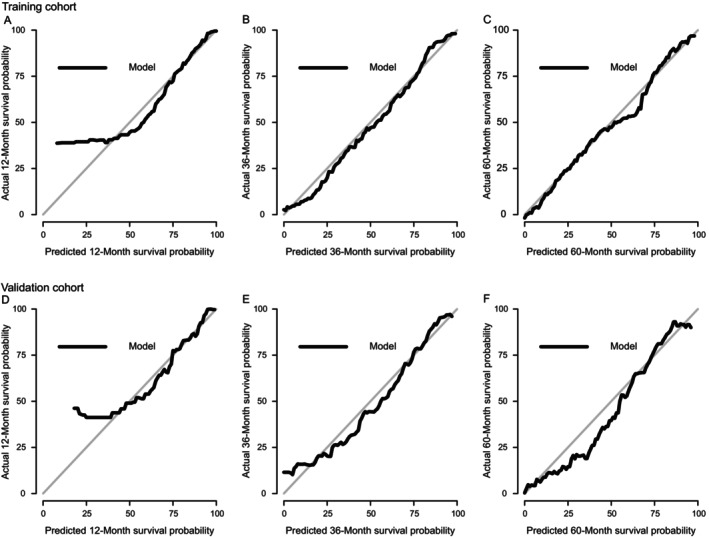
Calibration curves at various time points in the training and validation cohorts based on Coxm2. (A–C) Calibration curves to evaluate the effectiveness of the nomogram in predicting 1‐, 3‐, and 5‐year OS in the training cohort. (D–F) Calibration curves to evaluate the effectiveness of the nomogram in predicting 1‐, 3‐, and 5‐year OS in the validation cohort. OS, overall survival.

**FIGURE 5:**
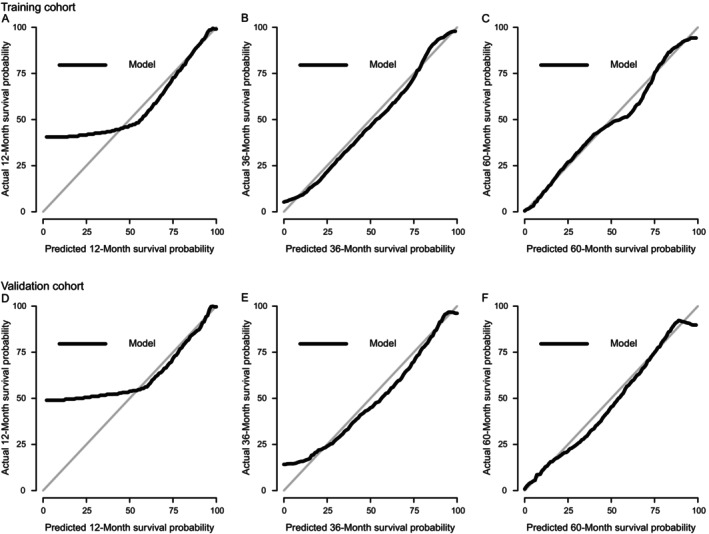
Resampling calibration curves at various time points in the training and validation cohorts based on Coxm2. (A–C) Calibration curves of 100 resamples to evaluate the effectiveness of the nomogram in predicting 1‐, 3‐, and 5‐year OS in the training cohort. (D–F) Calibration curves of 100 resamples to evaluate the effectiveness of the nomogram in predicting 1‐, 3‐, and 5‐year OS in the validation cohort. OS, overall survival.

The calibration curves at various time points in the training and validation cohorts based on Coxm2 are shown in Figure 4. The calibration curves of the 100 resamples are shown in Figure 5, which indicates that there was good consistency between the predicted survival rate and the actual survival rate at various time points.

### 3.6 Clinical Efficacy Analysis

**FIGURE 6:**
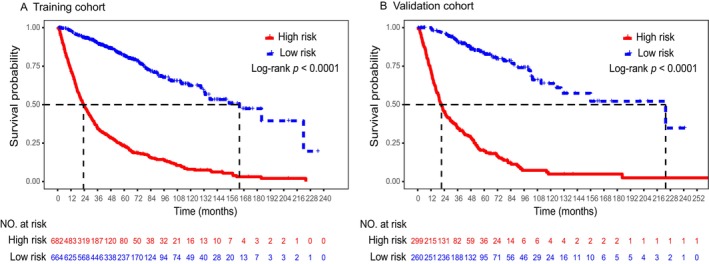
Based on the Coxm2 model, survival curves of patients with different risks were generated according to the mean score. (A) Survival curves of patients with different risks in the training cohort. (B) Survival curves of patients with different risks in the validation cohort.

To further evaluate the model's ability to discriminate prognosis, the prognostic risk scores of patients in the training and validation cohorts were calculated based on the Coxm2 model. Then, the patients were divided into low‐risk and high‐risk groups according to the mean score (274.29) in the training cohort. Survival curves for the different prognostic risk groups are shown in Figure 6. A total of 50.7% (682/1346) of patients in the training cohort and 53.5% (299/559) of patients in the validation cohort were at high risk. In both the training and validation cohorts, the prognosis of the high‐risk group was significantly worse than that of the low‐risk group (training cohort: HR, 6.932; 95% CI, 5.723–8.397; log‐rank *p* < 0.001; validation cohort: HR, 8.429; 95% CI, 6.180–11.497; log‐rank *p* < 0.001). The 1‐, 3‐, 5‐, and 10‐year survival rates were 72.5%, 34.4%, 23.1%, and 7.8% and 98.0%, 90.1%, 81.7%, and 62.2% (log‐rank *p* < 0.001) for the high‐risk and low‐risk groups, respectively, in the training cohort. The 1‐, 3‐, 5‐, and 10‐year survival rates were 71.9%, 34.6%, 19.8%, and 4.8% and 99.6%, 90.8%, 83.1%, and 62.0% (log‐rank *p* < 0.001) for the high‐risk and low‐risk groups, respectively, in the validation cohort. The median OS times for the high‐risk and low‐risk groups in the training cohort were 23.3 months (95% CI, 20.7–26.0 months) and 164.0 months (95% CI, 130.8–197.3 months), respectively. The median OS times for the high‐risk and low‐risk groups in the validation cohort were 21.5 months (95% CI, 18.4–24.6 months) and 223.7 months (95% CI, 100.0–347.3 months), respectively.

## 4 Discussion

Malnutrition significantly affects the prognosis of patients with cancers [27, 28, 29]. Our study suggests that more than 50% of patients have a high prognostic risk based on a predictive model constructed by nutritional indicators. Nutritional indicators, including albumin and prealbumin, were independent prognostic factors for OS. Our study also suggests that sex, age, baseline weight, food intake reduction grade, emerging disease, ECOG performance status, hospitalization frequency, clinical stage, hemoglobin suppression grade, platelet suppression grade, and liver function classification are significantly associated with OS. If screening was based solely on the NRS‐2002, only 37.5% of the patients in our study would have required nutritional intervention, but 50.7% (682/1346) of the patients in the training cohort and 53.5% (299/559) of the patients in the validation cohort had a high prognostic risk according to our model.

In our study, the established nomogram contained 13 clinically easy‐to‐obtain variables. The *C*‐indexes at 1, 3, 5, and 10 years in the training cohort were 0.848, 0.826, 0.814, and 0.799, respectively. The *C*‐indexes at 1, 3, 5, and 10 years in the validation cohort were 0.851, 0.819, 0.814, and 0.801, respectively. The model had great calibration in both the training and validation cohorts. Thus, the nutritionally high‐risk status according to the NRS‐2002 method prevents a large proportion of patients from receiving early nutritional intervention and does not provide prognostic information. Our study makes up for this deficiency. Our model included additional prognostic risk factors beyond those used in traditional nutritional screening. Early nutritional treatment can be carried out for high‐risk patients, and personalized condition assessments and nutritional treatment decisions can be made.

In our model, hunger, complications of chemoradiotherapy, and inflammatory indicators were combined to derive the actual nutritional status of patients and correlated them with clinical prognostics. For hunger, there are acute and chronic forms. A recent reduction in oral intake as a candidate variable for acute hunger and baseline weight and recent weight loss were considered as candidate variables for chronic hunger. Multivariable analysis showed that baseline weight and food intake reduction grade were independent prognostic factors. Reduced food intake in patients leads to muscle mass reduction and cachexia. The current weight loss grading methods do not take the potential benefit of a higher baseline weight into account in the risk assessment of patients with cancer or treatment‐related weight loss [30]. Patients with a higher baseline body weight may have more energy reserves, thus conferring a survival advantage [31].

The patients enrolled in the analysis were admitted to the hospital for antitumor therapy. The processes of radiotherapy and chemotherapy inevitably lead to a reduction in trilineage hemocytes. Patients with malnutrition lack raw materials for cell division [32], which limits the recovery of trilineage hemocytes. Thus, malnutrition would lead to a delay in antitumor therapy, extended treatment cycles, and ultimately a shortened survival time in patients [1]. In addition, due to the side effects of antitumor therapy such as chemoradiotherapy [1], patients often have impaired liver function, which leads to reduced protein synthesis or hypoproteinemia [33]. Those with complications such as hydrothorax or ascites have a worse prognosis. Due to the routine preventive application of leukopenia drugs, fewer patients delayed treatment due to leukopenia [34, 35, 36], so white blood cell suppression was not included in the model. In summary, hemoglobin suppression grade, platelet suppression grade, and liver function classification were included in the final model.

Inflammation is recognized as an increasingly important potential factor that increases the risk of malnutrition and may lead to an unsatisfactory effect of nutritional therapy and an increased risk of death [13]. Various forms of acute and chronic inflammation (complications caused by the primary disease, hospital‐acquired infection, and chemoradiotherapy) lead to increased catabolism and further malnutrition [37, 38]. These studies suggest that inflammation increases nutritional risk and that malnutrition further leads to a poorer prognosis, which is similar to our conclusion. In our study, CRP showed statistical significance in the univariable analysis but was not included in the model because it was not statistically significant in the multivariable analysis. PCT, which had too many missing values, was also not included in the model. Prealbumin and albumin are both nutritional and inflammatory indicators. In our enrolled patients, 53.9% had a prealbumin level that was lower than normal, and 59.6% had an albumin level that was lower than normal. Approximately half of the patients' prealbumin and albumin levels decreased below normal. Therefore, the mean score of the model was considered as the cutoff point. This may be in part why the model can stratify patient prognosis so well.

Studies have shown that acute or chronic inflammation of different degrees is closely related to the occurrence of malnutrition, and in diseases or injuries, metabolic and dietary intake changes occur [14, 39, 40]. There is a close interaction between inflammation and malnutrition. The existence of inflammation contributes to the development of malnutrition and limits the effectiveness of nutritional interventions [38, 41]. Similarly, malnutrition may reduce the effectiveness of drug therapy. Patients can benefit from early nutritional intervention, which may be due to its regulatory effect on systemic inflammation [19, 20]. Our study only identified inflammatory indicators as independent prognostic factors but could not determine the optimal intervention time for drug regulation. Inflammatory indicators are not limited to leukocytes, CRP, PCT, albumin, and prealbumin. Cytokines, especially IL‐1, IL‐6, and TNF‐alpha [20, 42], should also be collected and analyzed as candidate variables. Therefore, it is necessary to further design prospective interventional clinical trials to confirm our findings.

In summary, through Lasso regression, 13 key features crucial for the prognosis of patients with pan‐cancers were identified. Comparisons with the literature were made to validate the significance of these features and to explore their potential impact on clinical practice. First, some of these features have been validated in other studies, such as baseline weight and reduced food intake, indicating a significant effect of malnutrition on patient outcomes. Second, complications induced by chemotherapy and radiotherapy also affect patients' nutritional status and prognosis, emphasizing the importance of timely intervention. Finally, a comprehensive assessment of nutrition and inflammation status facilitates the development of personalized treatment plans, thereby improving patient survival rates and quality of life.

This study has several limitations. First, a prediction model based on pan‐cancer was constructed to explore the relationship between nutritional indicators and pan‐cancer prognosis. However, different types of cancer have different prognoses. In our study, no significant prognostic difference was found between different cancer types based on the site of the primary tumor. The main reason for this phenomenon is that patients tended to be enrolled at a later stage. A prognostic model based on nutritional indicators according to cancer type may be a better choice, but a sufficient sample size is needed for each cancer type. Second, this study did not consider possible differences in survival due to different treatment regimens. Third, despite all our efforts to improve the quality and quantity of the data, missing data were inevitable. However, since the proportion of missing data was low, the missing data were not imputed. Fourth, other nutritional indices such as Prognostic Nutritional Index (PNI), Controlling Nutritional Status (CONUT), Mini Nutritional Assessment (MNA), and Patient Generated Subjective Global Assessment (PG‐SGA) have not been assessed. Fifth, the comparison of nutrition at different stages was not explored to determine whether nutrition contributes to other symptoms or if the lack of nutrition itself constitutes a symptom. In future studies, the investigation of how nutritional indicators change as cancer progresses is necessary to ascertain the optimal timing for intervention.

## 5 Conclusion

A prognostic model for cancer patients based on nutritional indexes was developed. The model shows good discrimination in predicting OS. Thus, patients could be divided into different prognostic groups according to the mean score based on the nomogram. The nomogram performed well in internal validation. Further external validation is needed in future studies to determine its value in predicting prognosis.

## Author Contributions

**Zhi‐Rui Zhou:** conceptualization (equal), funding acquisition (equal), resources (equal), supervision (equal). **Lin Zheng:** data curation (equal), formal analysis (equal), investigation (equal), writing–original draft (equal). **Qian‐Qian Yu:** data curation (equal), formal analysis (equal), investigation (equal), writing–original draft (equal). **Wen‐Bin Ruan:** data curation (equal), formal analysis (equal), investigation (equal), writing–original draft (equal). **Jin Chen:** data curation (equal), formal analysis (equal), investigation (equal). **Qing‐Hua Deng:** resources (equal). **Ke Zhang:** resources (equal). **Xu‐Li Jiang:** resources (equal). **Wen‐Jun Jiang:** investigation (equal). **Dan‐Na Cai:** resources (equal). **Chen‐Jie He:** validation (equal), visualization (equal), writing–review and editing (equal). **Yu‐Feng Wang:** validation (equal), visualization (equal), writing–review and editing (equal). **Shen‐Li Jiang:** validation (equal), visualization (equal), writing–review and editing (equal). **Rui‐Zhi Ye:** conceptualization (equal), supervision (equal), validation (equal), visualization (equal). **Guang‐Xian You:** conceptualization (equal), funding acquisition (equal), resources (equal), supervision (equal). **Rong‐Biao Ying:** conceptualization (equal), funding acquisition (equal), resources (equal), supervision (equal).

## Conflicts of Interest

The authors declare no conflicts of interest.

## Ethics Statement

The study was approved by the Ethics Committee of Taizhou Cancer Hospital. Because this was a retrospective study, all patients were exempted from providing informed consent for the study.

## Data Availability

Datasets generated and analyzed during this study and the associated data dictionary and code are not publicly available. If readers wish to access the datasets analyzed and code utilized in this study, they need to email the corresponding author. The corresponding author will then make application to the Ethics Committee of Taizhou Cancer Hospital, and upon approval, they can provide the complete datasets and code.
